# Improving person-centred care for older persons with serious multimorbidity in LMICs

**DOI:** 10.4102/phcfm.v16i1.4440

**Published:** 2024-05-31

**Authors:** Duncan Kwaitana, Dorothee van Breevoort, Modai Mnenula, Kennedy Nkhoma, Richard Harding, Maya J. Bates

**Affiliations:** 1 Department of Family Medicine, School of Medicine and Oral Health, Kamuzu University of Health Sciences, Blantyre, Malawi; 2 Cicely Saunders Institute of Palliative Care Policy and Rehabilitation, Faculty of Nursing, Midwifery and Palliative Care, King’s College London, London, United Kingdom

**Keywords:** communication, multimorbidity, mentorship, elderly, palliative care, Africa, low- and middle-income countries

## Abstract

**Background:**

Few interventions are documented to meet person-centred needs of older people with serious multimorbidity in low- and middle-income countries where access to palliative care is limited. Most of the care in these settings is delivered by primary care health workers.

**Aim:**

This study reports the development and acceptability testing of a communication skills training and mentorship intervention for primary health care workers in Malawi.

**Setting:**

This study was conducted at Mangochi District Hospital in the south-eastern region of Malawi.

**Methods:**

Twelve primary health care workers (four clinical officers and eight nurses) working in the primary care clinics received the intervention. The intervention was designed using modified nominal group technique, informed by stakeholder interviews and a theory of change workshop. Acceptability is reported from thematic analysis of a focus group discussion with primary health care workers who received the intervention using NVivo version 14.

**Results:**

Older persons with serious multi-morbidity and their caregivers identified a need for enhanced communication with their healthcare providers. This helped to inform the development of a communication training skills and mentorship intervention package based on the local best practice six-step Ask-Ask-Tell-Ask-Ask-Plan framework. Primary health care workers reported that the intervention supported person-centred communication and improved the quality of holistic assessments, although space, workload and availability of medication limited the implementation of person-centred communication.

**Conclusion:**

The Ask-Ask-Tell-Ask-Ask-Plan framework, supported person-centered communication and improved the quality of holistic assessment.

**Contribution:**

This intervention offers an affordable, local model for integrating person-centered palliative care in resource-limited primary healthcare settings.

## Introduction

By 2050, 80% of the world population of older people will be living in low- and middle-income countries.^1^ Currently, older people and their families are underserved, with a high burden of physical and psychosocial symptoms and high formal and informal health service usage.^2,3^ According to the World Health Organization’s mortality projection analysis, serious health-related suffering is expected to increase in all regions by 2060. The most significant proportional rise is anticipated in low-income countries, with projections indicating a 155% increase between 2016 and 2060.^4^ These increases are primarily in diseases of ageing (cancer, cerebrovascular and lung diseases and dementia).

Only 12% of the 20 million people each year who require palliative care actually receive it, and while most provision is in high-income countries, 80% of need is in low- and middle-income countries.^5^ Although palliative care and pain relief are global human rights within existing covenants,^6^ palliative care is a neglected field in global health^7^ with woefully inadequate coverage. Strengthening primary health care is vital to improve outcomes and reduce inequity as all cadres assist in the provision of holistic person-centred care.^8,9^ Global palliative care development indicators have been aligned with the World Health Organization Operational Framework for Primary Health Care.^10^ Primary care utilisation for chronic disease management can improve patient outcomes and reduce costs.^11^

The African Palliative Care Association Standards for Providing Quality Palliative Care across Africa states that ‘effective communication, which meets the needs of the patient and their family, is essential in palliative care’.^12^ Communication skills, including shared decision-making, are vital for the delivery of person-centred care.^13^ Despite communication skills training approaches being developed for use in African settings,^14^ the ability to discuss issues around serious illness (‘breaking bad news’) remains challenging. Patients and families are left with significant gaps in their information needs.^15^ Fear of the emotional reaction by patients and family members to sharing difficult news about life-limiting illness has been reported as a barrier to the provision of palliative care by nurses in Ghana.^16^

Malawi is a low-income country in the south-central Africa. Over 80% of the population live in rural areas.^17^ Despite the significant health challenges posed by human immunodeficiency virus (HIV), tuberculosis (TB) and malaria, life expectancy rose from 55.6 to 64.7 years between 2010 and 2020.^17^ Primary health care is a priority for the government of Malawi as part of its commitment to provide Universal Health Coverage by 2030.^18^ Within this setting, there is limited literature reporting the experiences of older people in Malawi, many of whom live in poverty with significant burdens of need.^19^ ‘Home grown’ solutions to meet their needs are lacking.^20^ The aim of this study was to develop and test the acceptability of an intervention to support person-centred primary health care for older people with serious multi-morbidity.

## Research method and design

### Study setting

Research activities were nested within a larger three-country (Malawi, Ghana and Zimbabwe) study on primary palliative care. In Malawi, the study site was Mangochi District Hospital. This is a 500-bed government district hospital in the southern region of Malawi, staffed by 170 health care workers. It was selected as a major (government-funded) provider of primary health care services for the peri-urban and rural populations of Mangochi district (population 1.15 million).^17^ Although situated some distance away from Malawi’s capital and other main urban centres, research capacity exists at the hospital as it is a site for training programmes run by the Department of Family Medicine at the Kamuzu University of Health Sciences. In the clinical areas, non-doctor clinician cadres (clinical officers) and non-professional nurses (enrolled nurses) provide most primary health care (e.g. HIV, maternal and child health, non-communicable diseases [NCDs] and palliative care) at weekly clinics.

Convenience sampling was used to recruit older (>50 years) patient and caregiver dyads attending palliative care and/or NCD clinics and/or general outpatient clinics. The study consisted of two primary phases, denoted as Phase 1 and Phase 2, respectively, each featuring sub-phases designed to address specific thematic areas.

#### Phase 1: Intervention development

Phase 1 consisted of three sub-phases. In Phase 1a, stakeholder interviews were conducted with older patients and their caregivers. In Phase 1b, a Theory of Change (ToC) workshop was held, bringing together relevant stakeholders from various sectors of society. The ToC workshop delved into selected themes from the Phase 1a data and crafted a logic model for care pathways to improve patient care. The concluding Phase 1c involved the research team prioritising key elements from Phases 1a and 1b to inform the planning of interventions with primary care providers.

**Phase 1a - Stakeholder inverviews:** Verbal and written study information was given to participants. Written consent was provided by participants using either signature or thumbprint.

Between January and March 2021, 30 stakeholder interviews were conducted with 15 older patients (47% female) and 15 family caregivers (87% female). The age range of patients was 50–75 years (median 62 years) and of caregivers was 21–66 years (median 45 years). Serious multimorbidities included hypertension, diabetes, stroke, HIV and cancer (type not specified). The in-depth interviews were digitally recorded. Thereafter, simultaneous translation and transcription of data were conducted. Subsequently, the transcripts were imported into NVIVO 12 Pro, where qualitative data analysis was carried out.

**Phase 1b - Theory of change workshop:** In March 2022, 24 stakeholders (including older persons, members of the public, civil society representatives and government officials) were invited to a one-day meeting in Blantyre. Qualitative data from interviews with study participants were presented for discussion. Stakeholders were split into smaller groups where they discussed crucial aspects of care pathways aimed at enhancing patient care. Eventually, diverse viewpoints were synthesised, leading to the formulation of a process map for the primary care approach to the management of older people (see Appendix 1).

**Phase 1c - Modified nominal group technique:** Three virtual meetings were convened during September and October 2022 between members of the research team. The research team reviewed and discussed outputs from the stakeholder interviews and ToC workshop. Priorities for the intervention were ranked verbally. Subsequently, training strategies and materials were discussed, circulated and revised several times before reaching an agreement on the content and format of the intervention.

The agreed intervention comprised classroom teaching (two days) followed by eight weeks of mentorship incorporating observed consultations in the workplace, individual feedback, mentor support and group meetings (either virtual or in-person). The Ask-Ask-Tell-Ask-Ask-Plan framework was selected as the teaching model for communicating issues around serious illness. This framework had been previously adapted from Baile and Buckman’s six-step SPIKES framework for breaking bad news^21^ by one member of the team, following reflection on palliative care practice in Malawi. The Ask-Ask-Tell-Ask-Ask-Plan framework provides a named communication activity in each step, facilitating ease of recall in the work environment. Additional material for classroom training included an introduction to the principles of palliative care (holistic assessment and management of pain and other symptoms) and principles of communication (active listening skills, verbal and non-verbal communication). Mentorship comprised observed consultations and feedback, and group meetings. Mentors recorded observations at the end of each session.

#### Phase 2: Delivery of the intervention and acceptability testing

Phase 2 comprised two sub-phases, namely 2a and 2b. In Phase 2a, communication skills training and mentorship were introduced for primary care providers. Subsequently, in Phase 2b, the acceptability of the intervention was evaluated among mentees through a focus group discussion (FGD), coupled with documented observations of the mentors throughout the mentorship process.

**Phase 2a - Intervention delivery:** Health workers (enrolled nurses and/or clinical officers) with a minimum experience of 6 months providing primary health care services at either the palliative care or NCD clinics at Mangochi district hospital were purposively selected to receive the communication skills training and mentorship intervention. Study information was provided, and written consent was obtained from participants. Inclusion and exclusion criteria are listed in Table 1.

**TABLE 1:** Inclusion and exclusion criteria: Communication skills intervention with health care workers.

Inclusion criteria	Exclusion criteria
Clinical officers and/or nurses working in palliative care and/or non-Communicable Diseases clinics	Students and/or those newly qualified (whether nurses or clinical officers)
A minimum of 6 months working experience in the above clinics	Not willing to give written consent.

The communication skills teaching and mentorship intervention was delivered between November 2022 and February 2023 (see Table 2). Twelve health workers (eight enrolled nurses and four clinical officers, 30% male) received two days of classroom training, followed by eight weeks of mentorship from experienced members of staff.

**TABLE 2:** Intervention activities.

Activity	Total number	Number of clinicians and clinical officers receiving intervention		Number of enrolled nurses receiving intervention	
		Female	Male	Female	Male
Classroom training	1	4	3	8	1
Observed one-to-one patient consultation	12	4	3	8	1
Mentorship individual or paired feedback	12	4	3	8	1
Group online zoom mentorship sessions	3	-	2	6	1
WhatsApp group of mentors and mentees	Ongoing	4	3	8	1
Posters displayed in clinical areas	4	n/a	n/a	n/a	n/a

n/a, not applicable.

Posters displaying the six steps of the Ask-Ask-Tell-Ask-Ask-Plan framework were developed and displayed for reference in primary care clinical areas (see Figure 1).

**FIGURE 1:**
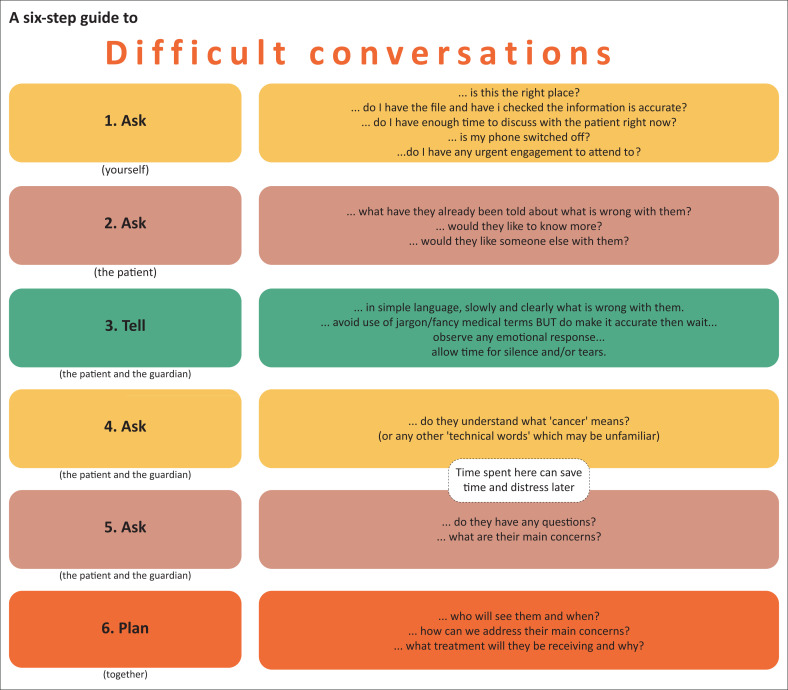
Ask-Ask-Tell-Ask-Ask-Plan framework for communication.

**Phase 2b - Acceptability of intervention: Focus group discussion and mentors’ observations:** Three months after the start of the intervention, an FGD was conducted by an independent research assistant. The FGD involved the primary care providers who had participated in the training and mentorship process. The length of the FGD was 90 min. It was conducted in both local language (Chichewa) and English and was recorded. Audio-recorded data were transcribed verbatim. Thematic analysis was conducted using NVivo version 14 by members of the research team. Themes were developed with reference to mentors’ observations through discussion amongst the wider research team.

### Ethical considerations

Ethical permission for this study was obtained from the College of Medicine Research and Ethics Committee, Blantyre, Malawi (approval number: P.08/20/3108) and Kings College London, London, UK (approval number HR-19/20-18524) on 08 October 2023. All participants provided written informed consent. Identification codes were assigned to participants and kept separate from all documents containing participant names. All electronic files were password protected with the rest of the paper-based documents kept in lockable cabinets and only accessible by the research team.

## Results

All 12 health workers received the intervention. Only eight health workers took part in the FGD (four were unavailable because of work commitments). The identified themes and subthemes are listed in Table 3.

**TABLE 3:** Communication skills and mentorship intervention acceptability themes and subthemes.

Themes	Subthemes
1. Design of intervention	1.1 Relevance to clinical practice in the workplace 1.2 Value of work-based mentorship 1.3 Duration and content of the training
2. Impact of intervention on person-centred care	2.1 Value of person-centred communication 2.2 Improved quality of holistic assessment 2.3 Improved treatment compliance
3. Challenges to implementing person-centred communication	3.1 Workload and space 3.2 Availability of medication 3.3 Language barrier

### Theme 1: Design of the intervention

#### Relevance to clinical practice in the workplace

Health care workers expressed that the training was relevant to their clinical practice. They discussed patient conditions and situations that are common among patients seen at palliative care and NCD clinics. In addition, they reported that the communication approach was applicable in other wards and clinics. Mentees expressed that they benefited from the intervention as they were able to apply a step-wise approach when conducting the consultation with an older person and their caregiver. The training helped them to approach the patients in a different way than they did before:

‘The training was a mind-set changer. It changed our attitudes unlike the way we used to think previously. We were taught that when a patient comes [*they*] should be approached through that strategy.’ (Mentee 5, 35–40 years age group, female, clinical officer)

#### Value of work-based mentorship

Respondents reported that the observed one-to-one clinic mentorship sessions allowed them to practice and improve the principles of breaking bad news and communication, especially where mentors were able to give feedback after the session on areas that needed to be improved:

‘It was so helpful because the time that we were doing [*the*] theory… we learnt about the strategy so when we went to practice it on real patients it was easy because we knew exactly that we have to do and whenever we did not do well, they were able to advise and guide us so it was helpful.’ (Mentee 6, 55–60 years age group, female, nurse)

#### Duration and content of the training

The mentees found the training content valuable, particularly its inclusion of additional topics such as pain and symptom assessment and management, which they believed enriched their skill set for patient care. They expressed overall satisfaction, emphasising the importance of dedicated time for hands-on practice of the acquired skills. However, they also conveyed a desire for extended practical and on-the-job training to further hone and apply the new skills effectively:

‘I feel like the training should be twofold. When we are done with theory then proceed to do practical on real patients to master the skill. Practicing on real patients helps because you may think that you’ve grasped the concept but actually you find that you’ve not. Clinical practical sessions on the actual patient is necessary.’ (Mentee 2, 60–65 years age group, female, nurse)

### Theme 2: Impact of the intervention on person-centred care

#### Value of person-centred communication

Respondents reported an increase in discussions with patients and families around disease and treatment following the intervention. This was thought to have improved disease understanding. The health care workers appreciated that person-centred communication can assist them to treat and support the patient:

‘[*T*]he patient is now aware of his or her disease, [*as well as being*] aware of the treatment. It’s a good feeling because most of the problems comes because the patient did not understand the treatment [*in*] the way he or she is supposed to take-up the medication but now we are taking time whereby we are in a position to explain more about the medications and the hospital visiting schedules.’ (Mentee 3, 40–45 years age group, male, nurse)

#### Improved quality of holistic assessment

Enhanced communication skills attributed to the intervention were reported to facilitate holistic assessments of older patients’ needs and those of their caregivers. Respondents reported that older people and their caregivers were willing to disclose more information about their situations. The findings showed that all participants expressed preference for person-centred care:

‘Most patients were just coming to the hospital with the mind that they are coming to receive medication but psycho-social issues were not being looked into. So, after the training, the patients are able to open-up and they are taking us as [*if we are*] one of their relative[*s*].’ (Mentee 2, 60–65 years age group, female, nurse)

This enabled the mentee to go further than only prescribing medication. One mentee reported that they arranged a home visit after the patient informed them about her situation at home:

‘We discovered that the patient lives with the mother who is the cervical cancer patient and was being discriminated against. She was isolated living alone in a separate dilapidated house …’ (Mentee 6, 55–60 years age group, female, nurse)

#### Improved treatment compliance

Mentees expressed that the communication skills they learned and practised helped them to communicate the importance of treatment adherence and the details of the medication schedule. Primary health care workers also recognised the role of the guardian in supporting the patient with taking the medication:

‘For me I’ve seen changes because at first it was like we were forcing the patients but now we do ask them about who has accompanied them and ask for permission if they can come to the consultation room in order to explain the treatment to both of them …’ (Mentee 6, 55–60 years age group, female, nurse)

### Theme 3: Challenges to implementing person-centred communication

#### Workload and space

Respondents reported that the small physical space in the consultation room was not adequate to provide person-centred care. Furthermore, they noted long queues of patients on clinic days. It was reported as tempting for the under-staffed providers to cut short holistic assessment and information sharing with patients in order to see all patients within a short period. This was felt to compromise quality care. However, it also encouraged the health care worker to improve patient flow in the hospital to minimise the queues:

‘Like in palliative care clinic, we try to deal with the queues; we divided the patients into groups, that this group should come on this particular day and this one on another day in order to reduce the queues. At first everyone was just coming in anyhow but as of now after the training we changed.’ (Mentee 5, 35–40 years age group, female, clinical officer)

#### Availability of medication

Respondents reported the challenge and frustration that, although they shared decision-making with patients and families, the medication was often not available. This felt like a waste of time for all concerned:

‘That’s another frustrating thing. You’ve spent quite a long time with the patient explaining things and he seem to understand everything about the treatment and at the end he finds that the medication is finished [*i.e. out of stock in the hospital pharmacy*]. The patient gets frustrated because had it been that he knew about that he’d have gone without spending much time.’ (Mentee 3, 40–45 years age group, male, nurse)

#### Language

The respondents expressed that some older people only spoke in the language of the local area (Chiyao) rather than using the national language (Chichewa). This was challenging for most health care workers who did not speak Chiyao. Mentees also expressed reservations when using Chichewa to explain some technical medical terms:

‘… For us to explain what cancer is in Chichewa it was difficult. When the patient is literate, it is not an issue but when is illiterate then it was a struggle.’ (Mentee 7, 30–35 years age group, female, nurse)

## Discussion

We report this study on the development and acceptability of a communication skills and mentorship intervention for primary health care workers in Malawi to support person-centred primary health care for older people with serious multimorbidity and their caregivers. Communication skills interventions for health workers working with older people have been published elsewhere in the literature^22,23,24^; however, to our knowledge, this is the first intervention to be reported from a primary health care setting in a low-income country. Primary health care workers in Malawi reported the relevance and value of the intervention to support person-centred care. Workload and space, medicine availability and language challenges were reported to limit their ability to apply the new skills.

Person-centred care for older persons gives ‘primacy to *understanding the person*’ and their unique interpretation and experience of illness or disability, in particular by taking a holistic view through recognition of psycho-social factors beyond presenting symptoms.^25^ This was reflected by older people and their caregivers in this study who reported financial needs (reduced costs of transport and medicine) and health system needs (improved care coordination and care delivered closer to home) as well as psycho-social needs (improved communication with health workers). The development of an intervention focusing on communication skills was informed in part by the priority of stakeholders but also driven by practical considerations within the context and timeframe of this study.

Priorities for enhanced communication are recognised as one of the pillars of person-centred care in the primary care space,^26^ through which ‘empowered people and communities’ receive ‘quality care’. Health care encounters around serious illness are typically conducted by generalist rather than specialist palliative care providers^27^ and, on the African continent, rely mainly on nurses, who constitute the largest number of trained health workers, as they provide leadership in primary care and palliative care.^28,29^ Given this situation, prioritising patients upon their arrival at the primary care setting could enable nurses to attend to more stable individuals, while clinical officers could focus on those presenting with complex symptoms. This approach aims to address challenges related to queuing.

O’Cathain et al. describe intervention development as ‘dynamic, iterative, creative, open to change and forward-looking to future evaluation and implementation’.^30^ Development of this intervention was creative, using a stakeholder-informed process and ToC logic model. Importantly for future implementation and scale-up, the intervention was delivered in (or as near as possible to) the primary health care clinical work environment and at minimal cost. In addition to supporting the time and some travel costs of mentors to the district site, intervention costs included venue hire and refreshments for a two-day training and the provision of mobile data for the mentees. The provision of an effective intervention at low cost is critical in low- and middle-income countries, to ensure the upskilling of primary health care workers at scale and to bridge the enormous gaps in access to primary palliative care.

The six-step SPIKES model (including Setting, Perception, Invitation, Knowledge, Emotions and Empathy, and Summary) has been widely reported and used for communication skills teaching in ‘breaking bad news’.^31^ Originally developed by oncologists in Canada, medical educators have proposed adaptations for different cultural settings.^32^ The modified six-step Ask-Ask-Tell-Ask-Ask-Plan framework is easy to recall, utilising basic written and/or spoken English terms. This is beneficial in settings where English is not a first language. The second ‘Ask’ of the framework allows for non-disclosure of information relating to illness and/or prognosis, as the patient is invited to determine what they know, what they want to hear and who should be present. Communication activities are proposed in a structured way in the framework, including four ‘Asks’, emphasising the need for (active) listening. The need for active listening has been highlighted in wider literature on nurse–patient interactions,^33^ although this may be lacking in Malawi where disrespect and abuse of maternity patients have been reported.^34^ Critically low staffing levels and burnout have previously been proposed as impacting the quality of routine primary care services.^35^ This was echoed in this study as primary health care workers reported excessive workload and suboptimal clinic space as barriers to the implementation of person-centred communication. Stockouts of medication also led to frustrations and a sense of time wasting. The intervention included one-to-one mentorship, including workplace observation and feedback, which was particularly valued by primary health care workers in this study. Mentorship provides a beneficial approach for improving the quality of primary health care, especially in low- and middle-income countries where the scarcity of health workforce is recognised.^36,37^

### Strengths and limitations

There are several limitations in this study. Intervention testing was conducted at a single site with 12 health workers in peri-urban or rural Malawi. Mentors were also part of the research team, which may have introduced bias. Malawi is one of only two African countries with advanced levels of access to palliative care.^5^ Adaptation of this intervention in other settings could be hampered by a lack of local skilled teachers and mentors. Nonetheless, data relating to suitable interventions to support person-centred care of older people in these settings are very limited,^19,20^ and this study goes some way to inform this critical gap. Because of time and resource constraints, this study failed to complete an extensive or comprehensive assessment of the effectiveness of the intervention. Future research in this area should prioritise perspectives of older people and their caregivers, for example, through detailed analysis of recordings of primary care consultations.^27^

## Conclusion

Stakeholder priorities informed the development of a communication skills training and mentorship intervention based around the local six-step Ask-Ask-Tell-Ask-Ask-Plan framework. Primary health care workers in Malawi receiving the intervention reported its relevance and value to the person-centred care of older people with serious multimorbidity and their caregivers. With increased population ageing, wider research documenting the needs and experiences of older persons with serious multimorbidity is vital. These needs will largely be the responsibility of primary health care providers in low- and middle-income countries, where few palliative care services exist. This communication skills training and mentorship intervention may be suitable for adaptation in other settings but more research is needed.

## Figures and Tables

**FIGURE 1-A1:**
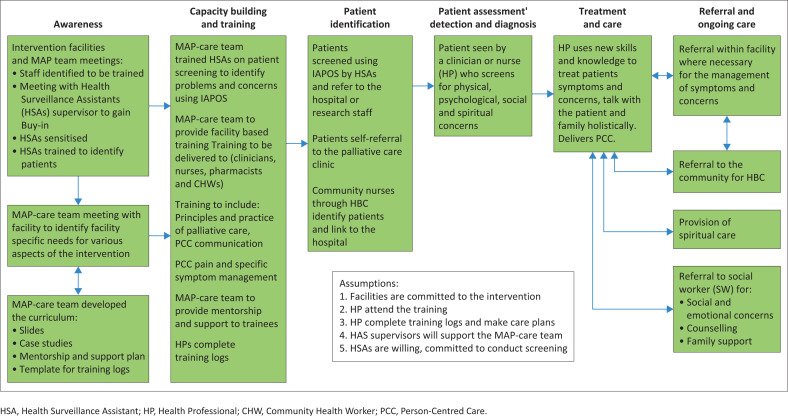
Process map for MAP-care integrated primary palliative care for older patients (≥ 50) with multimorbid illness (Malawi).
